# Updates on the Diagnostic Use of Ultrasonography Augmented With Perfluorobutane Contrast in Hepatocellular Carcinoma: A Meta-Analysis

**DOI:** 10.7759/cureus.60891

**Published:** 2024-05-23

**Authors:** Siraj Fahad Wally, Abdulaziz A Albalawi, Abdullah M Al Madshush, Maha Aljohani, Aysha J Alshehri, Faisal M Alamrani, Mariyah Alyahya, Farah S Aljohani, Areej Y Modrba, Rawan H Albalawi, Osama Y Albalawi

**Affiliations:** 1 Radiology, University of Tabuk, Tabuk, SAU; 2 Medicine, University of Tabuk, Tabuk, SAU; 3 General Practice, University of Tabuk, Tabuk, SAU; 4 Medicine and Surgery, University of Tabuk, Tabuk, SAU

**Keywords:** contrast-enhanced ultrasound, early cancer, hepatocellular carcinoma, liver cancer, systematic review

## Abstract

To investigate the diagnostic accuracy of contrast-enhanced ultrasound (CEUS) in the diagnosis of primary hepatocellular carcinoma (HCC), a thorough search was conducted for pertinent literature using PubMed, SCOPUS, Web of Science, Science Direct, and Wiley Library. This was a meta-analysis of diagnostic test accuracy. MetaDiSc 1.4 was used for all analyses and assessed statistical heterogeneity with the I2 index and the chi-square test. The random-effects model was applied where there was considerable heterogeneity. Using the eight elements of the Newcastle-Ottawa Scale (NOS) for cohort and case-control studies, we assessed the quality of the included studies. Our results included nine studies with a total of 2598 patients, and 1607 (61.8%) were males. The pooled overall sensitivity of perfluorobutane with CEUS was 85.6% (95% CI 0.832, -0.878, and P=0.000) and specificity was 91.5% (95% CI 0.899, -0.930, and P=0.000) with significant inter heterogeneity between studies (I2=94.3% and 95.7%), respectively. The pooled positive likelihood ratio was 12.42 (4.59 to 33.61, P=0.000). Our analysis revealed a symmetric summary receiver operating characteristic (SROC) curve and seven of the included studies are near the top left corner of the graph, indicating that this test has a high diagnostic value. The results showed that CEUS augmented with perfluorobutane contrast had good diagnostic accuracy (sensitivity and specificity) for primary HCC. Further real-world data studies are needed to confirm the good diagnosis accuracy of perfluorobutane CEUS in primary HCC.

## Introduction and background

Health-related cancer death is frequently caused by hepatocellular carcinoma (HCC), which is the sixth most common type of cancer globally [1,2]. Among the 6.35 million new instances of malignant tumors that are known to arise worldwide each year are an estimated 2.60,000 cases of primary hepatic carcinoma, or 4.1% of all cancers [3,4]. The incidence of HCC is still rising, and the prevalence is relatively high, especially in some developing countries where it is two to three times greater than in Western countries [5].

Patients often have advanced HCC upon diagnosis because the disease's symptoms are often not apparent in the early stages of the illness. Short disease duration and a dismal prognosis are the condition's hallmarks, and they have a detrimental impact on public health [6]. Early detection is one of the most important measures in both preventing HCC and increasing the survival rate of people who already have it. Thus, the development of a rapid, simple, and uncomplicated diagnosis method is required to facilitate the early identification and treatment of HCC.

The term contrast-enhanced ultrasound (CEUS) refers to the utilization of contrast-agent microbubbles alongside traditional ultrasonography to enhance visualization of differences in blood flow between surrounding tissue and a lesion. By incorporating CEUS, the ability to detect changes in blood flow associated with HCC can lead to more accurate diagnoses. Additionally, the distinct variations in blood perfusion between benign and malignant forms of HCC make CEUS a valuable tool for differentiation between the two forms of malignancy, CEUS can also be used as a diagnostic basis for differentiating between benign and malignant HCC [7,8]. It is also possible to do CEUS and unenhanced ultrasonography simultaneously. Thus, it is a workable method for the prompt and accurate diagnosis of HCC.

However, there is disagreement over the value of CEUS in the diagnosis of HCC. For example, it has been reported that a false positive diagnosis of HCC can be given to individuals with cholangiocarcinoma by CEUS [9]. Investigating the usefulness of CEUS with perfluorobutane in the diagnosis of primary HCC is the primary goal of this thorough analysis.

## Review

Methodology

Study Design and Duration

This was a meta-analysis of diagnostic test accuracy. Preferred Reporting Items for Systematic Reviews and Meta-Analyses (PRISMA) criteria are the subject of the current systematic review and meta-analysis [10]. PRISMA is an evidence-based minimum set of items for reporting in systematic reviews and meta-analyses. It consists of a 27-item checklist and a flow diagram, which outline the key elements that should be included in a systematic review or meta-analysis report. These items cover the study's objectives, methods, results, and conclusions, as well as potential biases and limitations. By following the PRISMA guidelines, systematic reviews and meta-analyses are transparent, reproducible, and comprehensive. This systematic review and meta-analysis were initiated in February of 2024.

Search Strategy

Relevant data was identified in articles using the following five primary databases: PubMed, SCOPUS, Web of Science, Science Direct, and Wiley Library. We searched just in English and took into account the unique requirements of each database.

Literature Search

To find the relevant studies, the following keywords were converted into PubMed Medical Subject Heading (MeSH) terms or topic terms in Scopus; "Hepatocellular carcinoma," "Liver cancer," "Liver tumour," "Hepatoma," "Perfluorobutane," "diagnosis," and " contrast-enhanced ultrasound." The necessary keywords were matched by the Boolean operators "OR," "AND," and "NOT". Among the search results were publications with full text in English, freely downloadable articles, and human trials.

Eligibility criteria for inclusion in this analysis included articles studying the effectiveness of CEUS with perfluorobutane in diagnosing primary HCC in adults (>18 years) of any study design, discussing required outcomes, involving only human subjects, written in English, and freely accessible. Exclusion criteria comprised case reports, unpublished data, reviews, letters, conference abstracts, and studies with insufficient data. Any disagreements in eligibility were resolved through discussion among the authors after the investigators completed their review.

Data Extraction

The search method's results were double-checked using Rayyan (Qatar Computing Research Institute (QCRI), Doha, Qatar) [11]. The investigators incorporated inclusion and exclusion standards into the combined search outcomes to assess the pertinence of the abstracts and titles. Every paper that satisfied the inclusion requirements was carefully read by reviewers. The writers discussed resolving disputes. The authorized study was uploaded using a previously created data extraction form. Data on the study titles, authors, year of the study, nation, participants, true positive (TP), false positive (FP), true negative (TN), and false negative (FN) were extracted by the authors. The risk of bias evaluation was made on a different page.

Strategy for Data Synthesis

A qualitative evaluation of the research findings and their component elements is given by the summary tables that were produced using data from pertinent studies. Once the data for the systematic review was collected, the optimal method for utilizing the data from the included study articles was selected.

Statistical Analysis

MetaDiSc 1.4 was used for all analyses. For statistical analysis, the following metrics were combined: pooled sensitivity, specificity, diagnostic odds ratio (DOR), positive and negative likelihood ratios (PLR and NLR), and the area under the summary receiver operating characteristic curves value (SROC). We assessed statistical heterogeneity with the I2 index and the chi-square test. The random-effects model was applied where there was considerable heterogeneity, as indicated by an I2 index > 50% and a Q-test p < 0.10; in other cases, the fixed-effects model was applied [12,13]. A p-value of less than 0.05 was deemed statistically significant. Because each meta-analysis had less than 10 trials, an investigation of potential publication bias was not carried out [13].

Risk of Bias Assessment

Using the eight elements of the Newcastle-Ottawa Scale (NOS) for cohort and case-control studies, we assessed the quality of the included studies. The NOS scale consists of eight things totaling nine points, divided into three dimensions. A low-quality study was one with a score of less than 4. A study was classified as medium-quality if it had a score of 4-6, and as high-quality if it received a score of >7. There is less chance of prejudice the higher the score [14].

Results

Search Results

**Figure 1:**
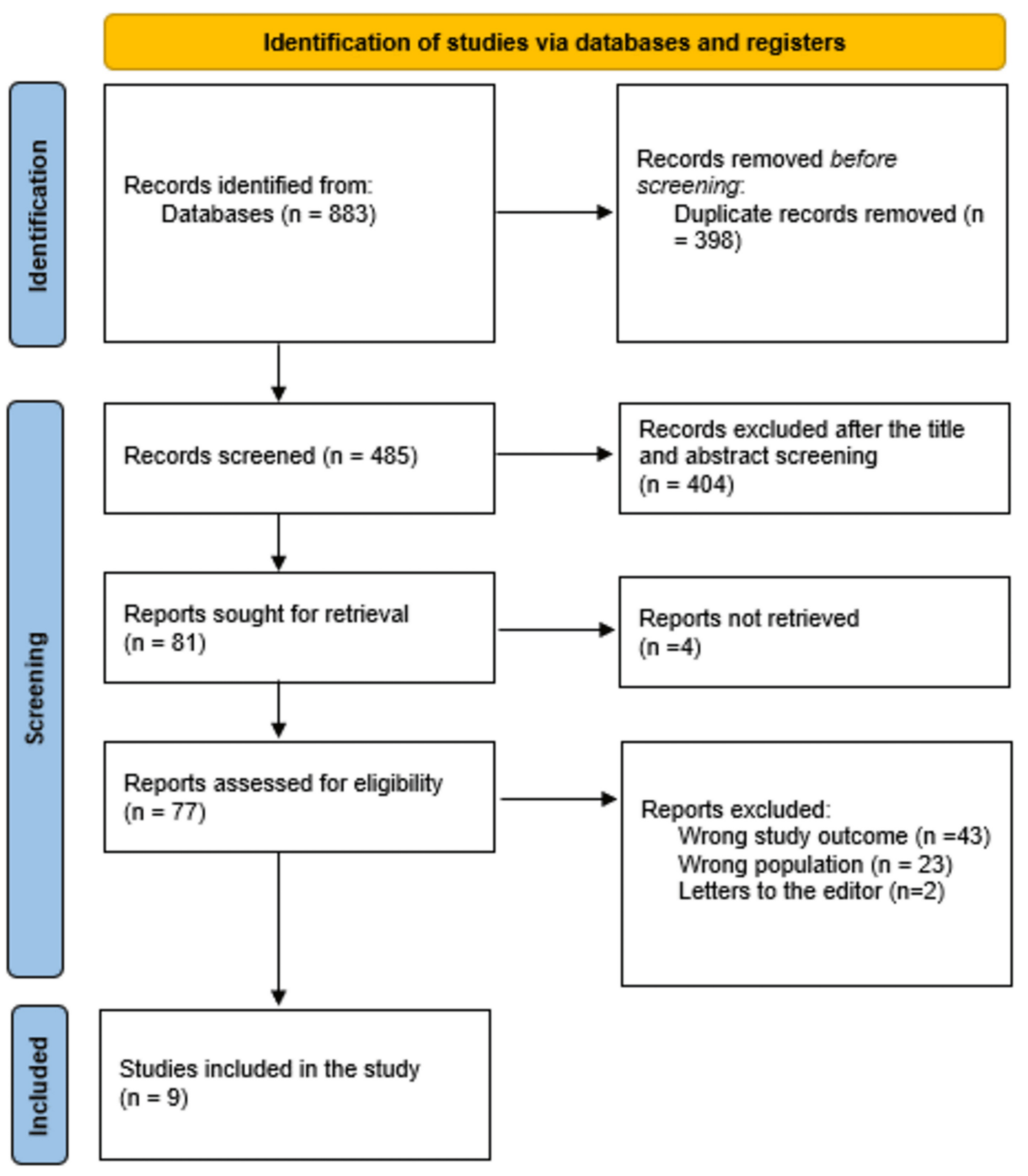
Study selection is summed up in a Preferred Reporting Items for Systematic Reviews and Meta-Analyses (PRISMA) flowchart.

After a thorough search, 883 study articles were found; 398 duplicates were eliminated. After 485 studies had their titles and abstracts screened, 404 were not included. Only four items out of the four reports that were requested were not found. After screening, 77 papers for full-text assessment, 43 were rejected due to incorrect study results, 23 were rejected due to incorrect population type, and two articles were editor's letters. This analysis contained nine acceptable research papers. An overview of the procedure used to choose studies is provided in Figure 1. 

The sociodemographic details of the research articles that are included are shown in Table 1. Of the 2598 patients in nine studies that made up our results, 1607 (61.8%) were men [15-23]. Four studies were retrospective in nature [8,15,17,20], four were prospective in nature [18,19,21,23], and one study was both retrospective and prospective [16]. Five studies were conducted in Japan [15-18,22], three in Korea [19,21,23], and one in China [20].

**Table 1:** Sociodemographic characteristics of the included participants. *NA=Not-applicable DN=dysplastic nodules, FNH= focal nodular hyperplasia, FP= false positive, FN=false negative, TP=true positive, TN=true negative, HCC=hepatocellular carcinoma, CEUS=contrast-enhanced ultrasound, NOS=Newcastle-Ottawa Scale, RCT=randomized controlled trial

Study	Study design	Country	Participants	Mean age	Males (%)	TP	FP	TN	FN	NOS
Hatanaka et al., 2008 [15]	Retrospective study	Japan	177 HCCs, 42 liver metastases, 20 liver hemangiomas, 6 DN and 4 FNHs.	68.8 ± 9.6	145 (67.9%)	171	4	6	68	7
Luo et al., 2010 [16]	Retrospective and prospective study	Japan	119	69.8 ± 6.5	67 (56.3%)	62	6	8	43	7
Shagdarsuren et al., 2016 [17]	Retrospective study	Japan	514	74	334 (64.9%)	396	0	40	18	6
Kudo et al., 2019 [18]	Prospective RCT	Japan	CEUS (n = 309) and B-mode US (n = 313)	65.7 ± 11.2	268 (43.1%)	28	11	0	270	NA*
Kang et al., 2020 [19]	Prospective study	Korea		65.6 ± 11	47 (79.6%)	34	0	9	16	6
Zhai et al., 2019 [20]	Preliminary study	China	65	55.1 ± 11.5	47 (72.3%)	11	1	2	19	7
Park et al., 2019 [21]	Prospective study	Korea	491	20-80	307 (62.53%)	12	4	4	471	8
Huang et al., 2018 [22]	Retrospective study	Japan	HCC (n = 311) non-HCC (n = 98)	53 ± 12.8	321 (78.5%)	66	40	65	213	7
kang et al., 2022 [23]	Prospective study	Korea	105	63 ± 11	71 (67.6%)	36	41	3	41	7

Diagnostic Accuracy

The pooled overall sensitivity of perfluorobutane with CEUS was 85.6% (95% CI 0.832, -0.878, and P=0.000) and specificity was 91.5% (95% CI 0.899, -0.930, and P=0.000) with significant inter heterogeneity between studies (I2=94.3% and 95.7%), respectively (Tables 2, 3).

**Table 2:** Diagnostic sensitivity of perfluorobutane with CEUS in primary HCC. Heterogeneity chi-squared = 139.25 (d.f. = 8), p = 0.000, Inconsistency (I-square) = 94.38, No. studies = 9 HCC=hepatocellular carcinoma, CEUS=contrast-enhanced ultrasound

Study	Specificity	95% Conf Interval
Hatanaka et al., 2008 [15]	0.966	(0.928-0.987)
Luo et al., 2010 [16]	0.886	(0.787-0.949)
Shagdarsuren et al., 2016 [17]	0.908	(0.877-0.934)
Kudo et al., 2019 [18]	1	(0.877-1.000)
Kang et al., 2020 [19]	0.791	(0.640-0.900)
Zhai et al., 2019 [20]	0.846	(0.546-0.981)
Park et al., 2019 [21]	0.75	(0.476-0.927)
Huang et al., 2018 [22]	0.504	(0.415-0.592)
Kang et al., 2022 [23]	0.923	(0.791-0.984)
Pooled Sen	0.856	(0.832-0.878)

**Table 3:** Diagnostic specificity of perfluorobutane with CEUS in primary HCC. Heterogeneity chi-squared=184.58, (d.f.=8) p=0.000, Inconsistency (I-square)=95.7%, No. studies=9 HCC=hepatocellular carcinoma, CEUS=contrast-enhanced ultrasound

Study	Specificity	95% Conf Interval
Hatanaka et al., 2008 [15]	0.944	(0.846-0.985)
Luo et al., 2010 [16]	0.878	(0.752-0.954)
Shagdarsuren et al., 2016 [17]	1	(0.815-1.000)
Kudo et al., 2019 [18]	0.961	(0.931-0.980)
Kang et al., 2020 [19]	1	(0.794-1.000)
Zhai et al., 2019 [20]	0.95	(0.751-0.999)
Park et al., 2019 [21]	0.992	(0.979-0.998)
Huang et al., 2018 [22]	0.842	(0.971-0.885)
kang et al., 2022 [23]	0.5	(0.387-0.613)
Pooled Spe	0.915	(0.899-0.930)

**Figure 2:**
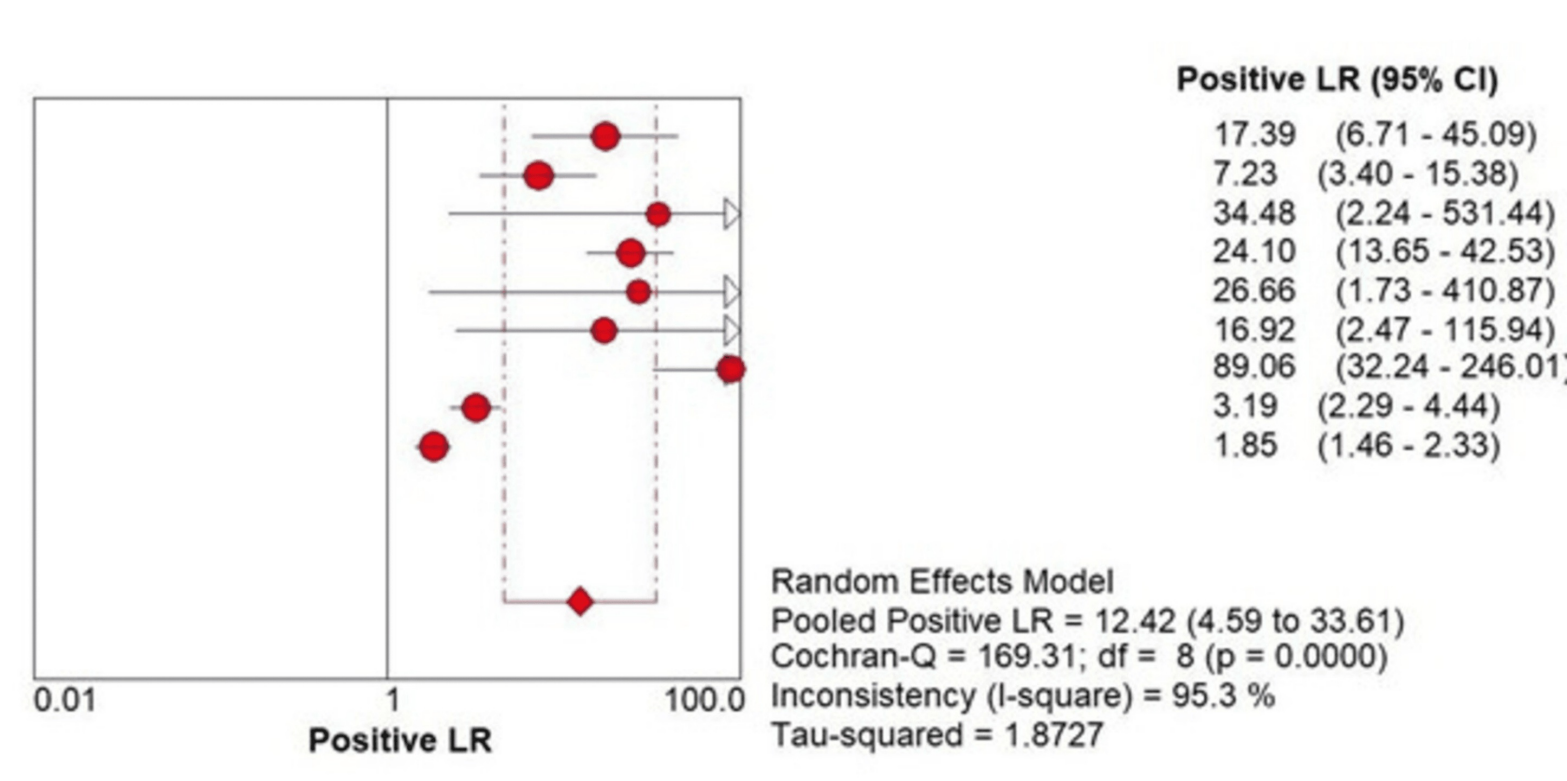
Positive likelihood ratios (LR) of perfluorobutane with CEUS in primary HCC. HCC=hepatocellular carcinoma, CEUS=contrast-enhanced ultrasound

**Figure 3:**
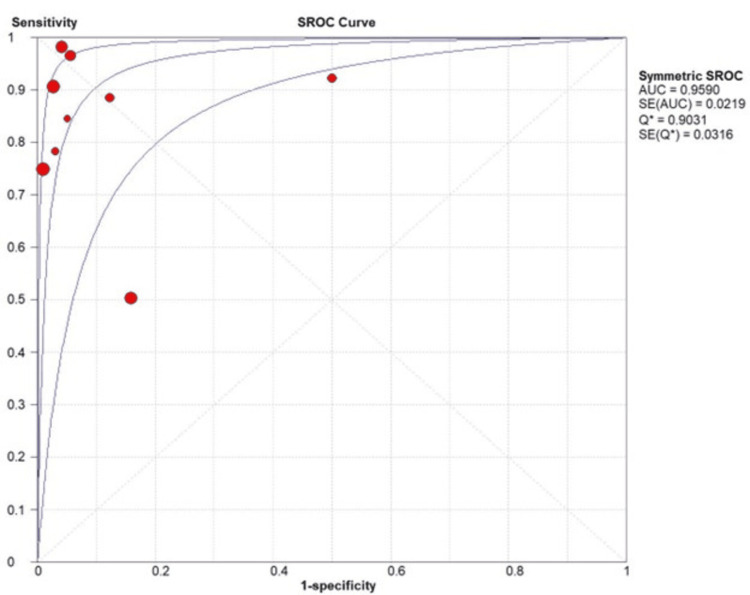
Summary Receiver Operating Characteristic (SROC) Curve Plot for Moses-Shapiro-Littenberg Method.

The pooled positive LR was 12.42 (4.59 to 33.61, P=0.000) (Figure 2). Our analysis revealed a symmetric SROC curve and seven of the included studies are near the top left corner of the graph, indicating that this test has a high diagnostic value (Figure 3).

Discussion

The clinical fatality rate of HCC, a malignant tumor, is significant. As a result, there are still many grave concerns regarding the worldwide prevention and treatment of HCC. Residents' health and quality of life are at risk due to the rising public health issue of HCC [24,25]. Numerous investigations have demonstrated that HCC progresses more quickly than other malignant tumors [26,27]. This has to do with the liver receiving blood from both the portal vein and the hepatic artery [26].

We demonstrated high sensitivity and specificity of 85.6% (95% CI 0.832, -0.878, and P=0.000) and 91.5% (95% CI 0.899, -0.930, and P=0.000), respectively. Which was higher than the reported values by Peng et al. [28] 0.72 for sensitivity and 0.92 for specificity assessing the diagnostic values of CEUS in HCC. When it comes to advanced-stage Barcelona Clinic Liver Cancer (BCLC), however, the five-year survival rate is 39.0% [20]. According to the American Society of Hepatology and the European Society of Hepatology recommendations, dynamic contrast-enhanced CT is one of the standard tests for suspected liver cancer, with a sensitivity and specificity of 71% and 87%, respectively; needle biopsy of liver mass usually fails for the purpose of diagnosis; tumour cells can be seen in the pathological tissue of liver mass injury biopsy; the diagnostic accuracy of tumour diameter >2 cm is greater than that of diameter 1-2 cm and diameter <1 cm [29]. Dynamic contrast-enhanced CT is sometimes used to evaluate individuals with strong suspicions, but because of its high cost and lack of specificity, it is not recommended for screening due to its high cost and radiation risks [30,31].

Higher priced pages, however, restrict its use in liver cancer screening. Magnetic resonance imaging (MRI) is a diagnostic tool that can be used to identify abnormalities that other means of diagnosis are unable to detect, and it is accurate enough to identify liver cancer [32].

There is a noticeable amount of variability in our analysis, which could introduce bias and affect the outcomes [33]. Given the potential impact of nodule size and variations on the ultimate staging, we conducted grouped subgroup analyses. Regretfully, we can only reduce heterogeneity, despite having taken into account possible causes and hoping to eradicate it through subgroup analysis. One significant factor is that individuals are essential to the operation and interpretation of the images produced by CEUS. Even with the same lesion, various radiologists' backgrounds and skills could produce differing findings.

The ultrasonography contrast agent perfluorobutane is a very specific mononuclear phagocyte ultrasonography contrast agent. It is distributed in China and Japan. Hydrogenated phosphatidylserine sodium, which can be metabolised by the kidney and liver, makes up the microsphere shell, whereas the upper filling is colourless perfluorobutane gas that can be broken down by pulmonary respiration. One of its common uses at the moment is the diagnosis of liver cancer.

## Conclusions

The results of the study demonstrated that CEUS enhanced with perfluorobutane contrast exhibited promising diagnostic accuracy, as evidenced by favorable sensitivity and specificity values for primary HCC. However, to further validate and consolidate these findings, additional real-world data studies are essential. These future studies will play a crucial role in confirming the robust diagnostic accuracy of perfluorobutane CEUS in the context of primary HCC, thereby enhancing its clinical utility and reliability in the diagnosis of this condition.
